# Risk factors for treatment interruption and severe adverse effects to benznidazole in adult patients with Chagas disease

**DOI:** 10.1371/journal.pone.0185033

**Published:** 2017-09-26

**Authors:** Mario J. Olivera, Zulma M. Cucunubá, Carlos A. Valencia-Hernández, Rafael Herazo, Diana Agreda-Rudenko, Carolina Flórez, Sofía Duque, Rubén S. Nicholls

**Affiliations:** 1 Red Chagas Colombia, Bogotá DC, Colombia; 2 Grupo de Parasitología, Instituto Nacional de Salud, Bogotá DC, Colombia; University of Oxford, UNITED KINGDOM

## Abstract

**Background:**

Etiological treatment of Chagas disease in chronic asymptomatic patients is still in debate and the adverse effects of traditional drugs are one of the main concerns in clinical practice. This study evaluated retrospectively the safety profile of benznidazole (BZN) and identified predictive factors for definite treatment interruption and development of severe reactions in adult patients treated with BZN in Colombia.

**Methods:**

Retrospective follow-up study conducted by review of medical records of adults with chronic Chagas disease treated with BZN in Colombia. A parametric survival analysis based on a generalized gamma distribution was used for assessing risk factors for treatment interruption. A multinomial logistic regression model was used to estimate the probability of severe adverse drug reactions (ADRs). Statistical associations were expressed as time ratios (TR) and adjusted odds ratios (aOR) respectively.

**Results:**

In total 224 adults patients treated with BZN were included; 172 (76.8%) completed the standard therapy (60 days of treatment), 205 (91.5%) presented ADRs and 52 cases (23.2%) required treatment interruption. The predominant symptoms were: rash (37.9%), itching (33.7%), epigastric pain (26.4%), abdominal bloating (24.2%) and nausea (22.1%). ADRs were mild (57.4%), moderate (35.5%) and severe (7.3%). Time to treatment interruption was significantly shorter when using doses of BZN ≥ 6 mg/kg/day (TR 0.55; 95% CI 0.39–0.76), presenting severe ADRs (TR 0.12; 95% CI: 0.07–0.19) and eosinophilia (TR 0.68; 95% CI: 0.49–0.94). Female sex (aOR 3.98; 95% CI 1.56–10.16), dose of BZN ≥ 6 mg/kg/day (aOR 1.41; 95% CI 1.17–1.70) and presence of > 3 ADRs (aOR 6.47; 95% CI 1.24–34.34) were considered as risk factors for developing severe ADRs.

**Conclusions:**

Dose, severity of ADRs, eosinophilia and female sex were the main predictors for treatment interruption or severe ADRs. The potential implications of these findings are discussed.

## Introduction

Despite decades of control efforts, Chagas disease remains one of the principal tropical diseases in the Americas. The World Health Organization estimates that this disease affects 6–7 million people worldwide and it causes more than 7,000 deaths per year [1]. It represents one of the largest parasitic disease burden with 806,170 disability adjusted life years in Latin-America [2]. Currently, anti-parasitic treatment options for Chagas disease are limited by only two old anti-parasitic drugs, benznidazole (BZN) and nifurtimox [3]. A recent study has determined a lack of efficacy of BZN once the chronic heart complications are already established [4]. Even though, the clinical implications of aetiological treatment are still in debate for asymptomatic cases, a parasitic effect has been consistently proved in this population [5]. The current trend in the majority of countries is to treat cases with Chagas disease in asymptomatic stage. The expected benefits of aetiological treatment include a reduction in the incidence of chronic complications and death [6–8].

Adverse drug reactions (ADRs) associated with BZN are one the main disadvantages of using this drug [9]. In some adult populations the incidence of ADRs has reached up to 100% [10] leading to interruption of treatment in up to 31.6% of patients frequently due to cutaneous reactions [11]. The most common ADRs observed with BZN have been dermatological alterations followed by gastrointestinal disturbances [9]. Children seem less susceptible to ADRs than adults [12]. Some factors have been proposed as predictors of interruption to treatment including female sex [13] and the number of ADRs [14]. However, there are few studies that support these findings in different populations. Due to the high incidence of ADRs associated with BZN, it is important to have tools to identify patients at risk of developing ADRs and interrupting treatment, particularly in endemic countries.

This study aimed at evaluating the tolerance and safety profile of BZN in a cohort of adult patients with chronic Chagas disease in Colombia and identifying the risk factors for definitive treatment interruption and severe ADRs.

## Materials and methods

Observational and retrospective study of a cohort of patients with chronic Chagas disease treated with BZN at the Colombian National Health Institute (Instituto Nacional de Salud) from June 2000 through December 2013. We follow the recommendations of the Strengthening the Reporting of Observational studies in Epidemiology-STROBE (S1 File).

### Patients and procedures

Patients were asked for permission for accessing their clinical records. The selection criteria were: i) Age ≥18 years old ii) Confirmed as positive to *Trypanosoma cruzi* infection by two serological tests, using both ELISA and immunofluorescent antibody (IFAT) tests following international recommendations [15], iii) History of having received treatment with BZN at the National Health Institute iv) Evidence of complete clinical records (pre, during and post-treatment). Patients under 18 years old and patients who had not attended at least one clinical control during their treatment were excluded (5 patients).

The treatment regime, according to the National Treatment Guidelines in Colombia [16], dosing of BZN (100 mg tablets) consisted of 5–7 mg/kg/day divided into two doses daily for 60 days and a clinical follow-up at the start, middle and end of the treatment. Clinical evaluation included biological analyses (complete blood count, liver and renal function tests) at 0, 20, 40, and 60 days. Drugs were provided to the National Health Institute by the Ministry of Health and Social Protection of Colombia.

As part of the follow-up protocol, during each control patients were asked about the occurrence of expected ADRs and specific forms for adverse drug reactions were filled by doctors during each consultation reporting the results of laboratory tests and the presence of signs and symptoms. Specific ADRs were medically treated according to their severity; mild and moderate events were treated firstly with medications according to specific symptoms (analgesics for headache, proton pump inhibitors for dyspepsia, anti-H2 for allergies). Temporary treatment suspension was implemented for a few days when symptomatic medications did not work and then the drug scheme was continued starting with a low dose that was gradually increased to reach the original prescribed dose [16].

The records were manually revised and causality of adverse reactions was determined by using the Naranjo algorithm [17], then double checked and the agreement was estimated. A consensus was made between the investigators when disagreement occurred. The severity of the ADRs was classified according to the level of affectation of the normal life of the patient. This scale allowed for categorical classification of ADRs as mild, moderate, severe or fatal [9,18].

### Statistical methods

Means, standard deviations, medians and proportions were used for presenting descriptive results for continuous and categorical variables respectively. The chi-square or Fisher’s exact test, as appropriate, and Student´s t test were used to compare categorical and continuous variables. For causality measures, through Naranjo Algorithm, the test–retest reliability was tested by using the kappa statistic (k). The kappa coefficient was interpreted as proposed by Landis and Koch [19].

Risk factors for Treatment Interruption: Time-to-event (time-to-interruption) was defined as the time in days from the start of treatment until definite interruption due to ADRs. A Cox proportional hazards model was considered not suitable, given that a semi-parametric proportional hazards assumption was not fulfilled [20]. Therefore, a parametric survival model was chosen for the survival analysis, and then the exponential, Weibull, log-logistic, log-normal and GG distributions were compared [21]. Akaike Information Criterion’s lowest value was considered as the best fit to data [22,23]. Times Ratios (TR) were used to assess risk factors for treatment interruption; a TR > 1 implies that the variable is associated with a longer time from the start the treatment with BZN until its interruption [21].

Risk factors for Severe ADRs: a multinomial logistic regression model was used to estimate the probability of developing severe ADRs. The Hosmer and Lemeshow test was used to show how adequately the model fits the data [24]. Associations were estimated with Odds ratios (OR) and their respective 95% confidence intervals (CI).

All the statistical analyses were performed using the Stata® (release 11.0) software package (Stata, College Station, TX) and R statistical package (R Development Core Team, 2008, Vienna, Austria)[25]. P-values < 0.05 were considered as statistically significant.

### Ethical considerations

The study protocol was approved by the Technical Research Committee and Ethics Research Board at the National Health Institute in Bogotá, Colombia: protocol CTIN-014-11, Minute 9 of December 11, 2012. Participation was voluntary and patients were asked for informed written consent to access information on their clinical records.

## Results

### General findings

From 2000–2013, 420 records of adult patients with chronic Chagas disease were identified. Of these, 106 patients had not received etiological treatment, 85 had been treated with nifurtimox and 229 with BZN. Of the 229 patients treated with BZN, five were excluded because they did not complete medical record.

In total, 224 patients were treated with BZN of whom 172 (76.8%) completed 60 days of treatment and 52 (23.2%) required definitive treatment interruption due to intolerance to BZN. The total dose of BZN ranged from 200 to 600 mg/day (mean 362.3 ± 66.7) and the duration of treatment ranged from 3 to 75 days (mean 51.6 ± 16.3). Basic demographics and clinical characteristics at baseline are shown in Table 1.

**Table 1:** Proportion of adverse drug reactions to benznidazole stratified by baseline characteristics and events (interruption of benznidazole).

Variable	Category	ADRs	
		Yes, n (%)	No, n (%)
Sex	Female	111 (91.0%)	11 (9.0%)
	Male	94 (92.2%)	8 (7.8%)
Age	18–50 years	121 (91.7%)	11 (8.3%)
	>50 years	82 (91.1%)	8 (8.9%)
Interruption of treatment	Yes	52 (100%)	0 (0%)
	No	153 (88.9%)	19 (11.1%)
Total		205 (91.5%)	19 (8.5%)

ADRs: Adverse Drug Reactions.

Of the 224 patients treated, 205 (91.5%) patients had at least one ADR during the course of the treatment with BZN and 181 (80.8%) had ≥ 3 ADRs; only nine patients (4.0%) had only one ADR. All ADRs disappeared after discontinuation of treatment.

Specific reported symptoms in order of frequency were: rash 107 (37.9%), itching 73 (33.7%), epigastric pain 61 (26.4%), abdominal bloating 56 (24.2%) and nausea 51 (22.1%), as shown in Table 2.

**Table 2:** Frequency of adverse drug reactions to benznidazole according to systems or organs.

Systems or organs	Absolute Frequency	%	Symptom/alteration	Frequency	
				n	%
Skin	282	39.4	Rash	107	37.9
			Itching	95	33.7
			Urticaria	23	8.2
			Angioedema	21	7.4
			Photosensitivity	15	5.3
			Bullous eruption	12	4.3
			Skin peeling	9	3.2
Gastrointestinal	232	32.3	Epigastric pain	61	26.4
			Abdominal bloating	56	24.2
			Nausea	51	22.1
			Abdominal pain	42	18.2
			Vomiting	8	3.5
			Diarrhoea	6	2.6
			Constipation	5	2.2
			Dysphagia	3	1.3
Body as a whole—general disorders	51	7.1	Headache	19	37.3
			Adynamia	15	29.4
			Asthenia	14	27.5
			Fever	3	5.9
Autonomic nervous system	45	6.3	Loss of appetite	31	68.9
			Increase in appetite	14	31.1
Haematological	37	5.2	Eosinophilia	23	62.2
			Lymphocytosis	8	21.6
			Neutrophilia	4	10.8
			Leukopenia	2	5.4
Musculoskeletal	30	4.3	Myalgia	17	56.7
			Arthralgia	13	43.3
Central and peripheral nervous system	26	3.6	Sleep disturbance	14	53.8
			Depression	5	19.2
			Paraesthesia	4	15.4
			Vertigo	3	11.5
Liver	13	1.8	Increased (ALT) levels	7	53.8
			Increased (AST) levels	6	46.2
**TOTAL**	**717**	**100**			

The symptoms leading to definitive interruption of treatment in order of frequency were: rash 11 (21.2%), urticarial reaction 8 (15.4%), angioedema 7 (13.5%), skin peeling 6 (11.5%), epigastric pain 6 (11.5%), bullous eruption 5 (9.6%), paraesthesia 3 (5.8%), drug reaction with eosinophilia and systemic symptoms 2 (3.8%), sleep disturbance 2 (3.8%), depression 1 (1.9%) and three times increase in baseline levels of alanine aminotransferase 1 (1.9%).

The specific analysis of differential risk for each ADR showed that the risk of presenting dermatological ADRs increases when using doses of BZN ≥ 6 mg/kg/day. Significant differences were seen in rash (OR 1.93; 95% CI 1.22–3.04), itching (OR 2.33; 95% CI 1.42–3.84), urticaria (OR 4.77; 95% CI 1.67–13.64), angioedema (OR 2.84; 95% CI 1.16–6.93), photosensitivity (OR 2.93; 95% CI 1.10–7.72), bullous eruption (OR 3.36; 95% CI 1.15–9.81) and skin peeling (OR 4.93; 95% CI 1.13–21.52).

### Causality and severity of ADRs

In this study, overall 716 ADRs were identified (Table 2). According to the Naranjo algorithm, the ADRs were classified as follows: 193 (26.7%) as definitive, 180 (25.1%) as probable, 174 (24.3%) as possible and 169 (23.6%) as doubtful. The intra-observer kappa for assigning causality was k = 0.91 (95% CI: 0.90–0.92). In terms of severity, 411 (57.4%) cases were classified as mild, 253 (35.3%) as moderate and 52 as severe (7.3%) and no fatal cases were reported. The intra-observer kappa for assigning severity to BZN was k = 0.96 (95% CI: 0.95–0.98)

### Risk factors for treatment interruption

The average time to interruption was 26.7 days (95% CI: 22.3–34.6 days). The bivariate analysis showed a statistically significant shorter time of treatment for the presence of severe ADRs (p< 0.0001), dose (p = 0.029), eosinophilia (p = 0.038) and sex (p = 0.007).

**Fig 1:**
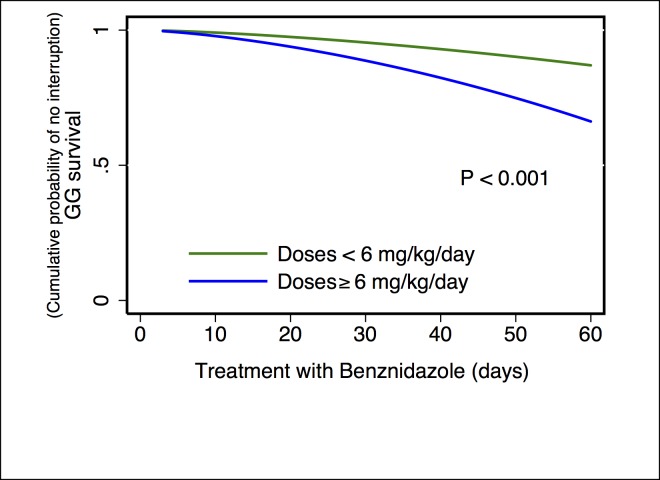
Generalized Gamma (survival) curves for risk factors associated with time free to treatment interruption: dose.

**Fig 2:**
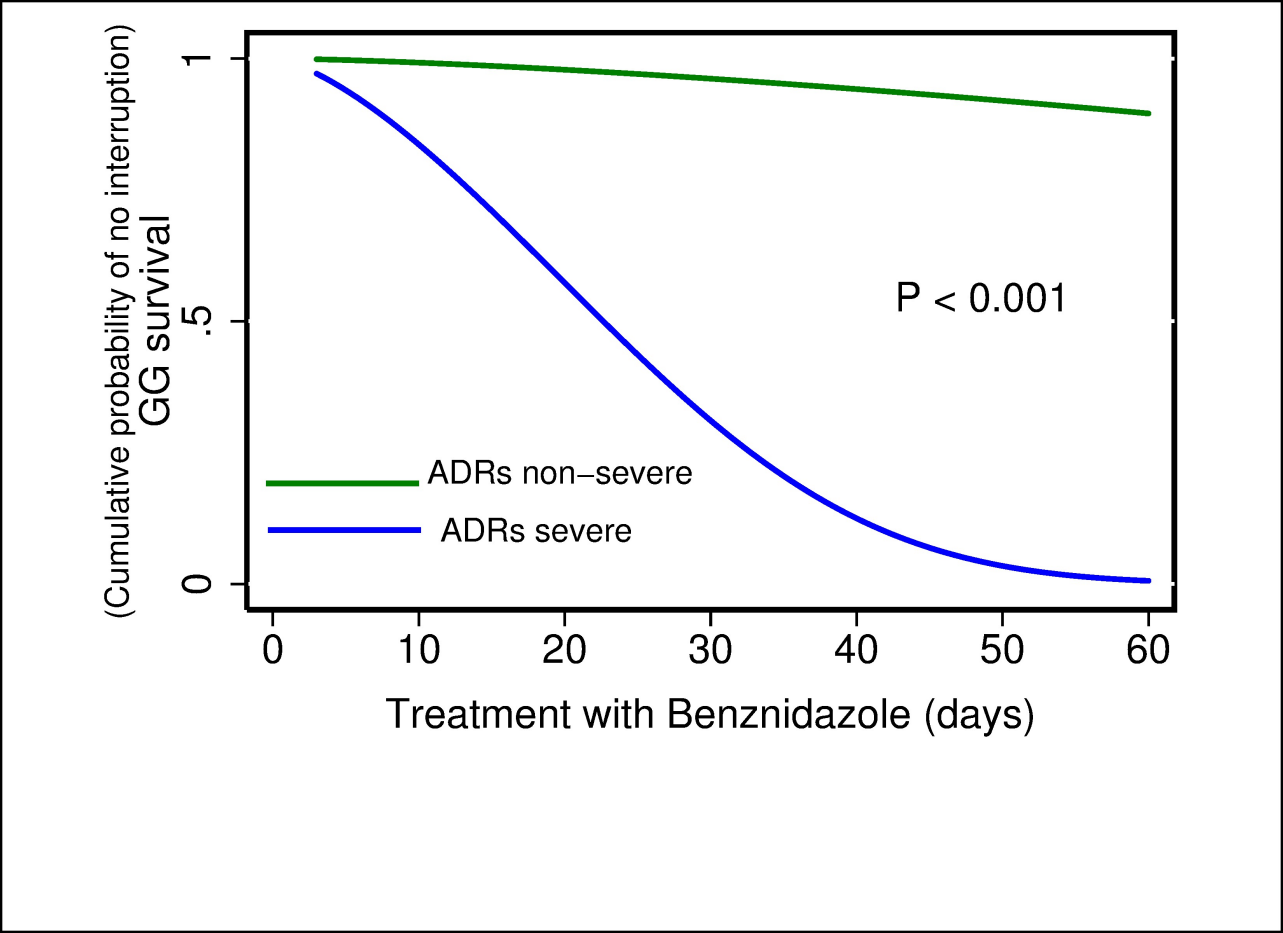
Generalized Gamma (survival) curves for risk factors associated with time free to treatment interruption: severity of adverse drug reactions (ADRs).

**Fig 3:**
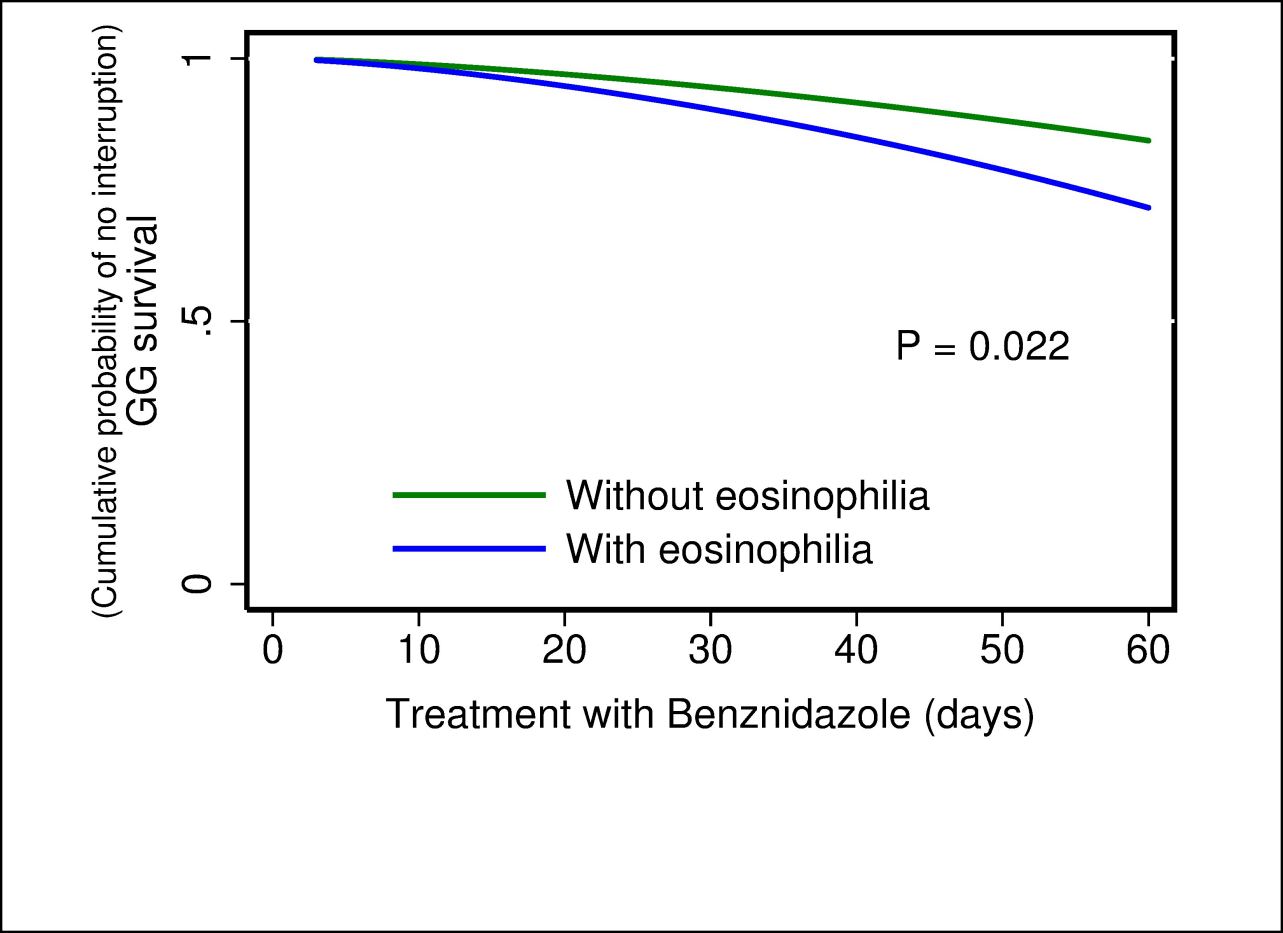
Generalized Gamma (survival) curves for risk factors associated with time free to treatment interruption: eosinophilia.

**Fig 4:**
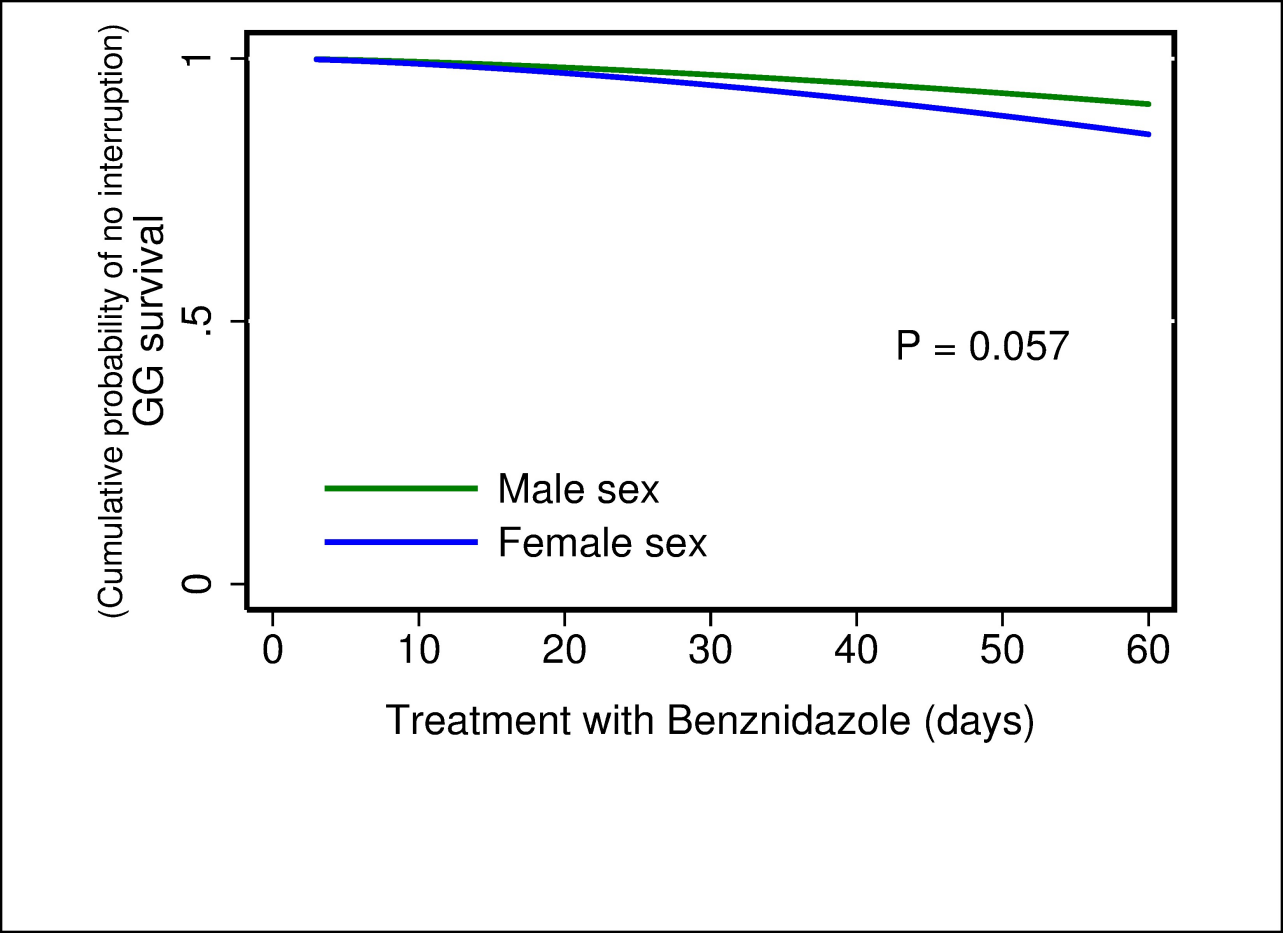
Generalized Gamma (survival) curves for risk factors associated with time free to treatment interruption: sex.

For the multivariate analysis, the results of the comparison between parametric models show that the Generalized Gamma distribution was the best candidate according to the AIC value (347.8) compared with the others: exponential (350.2), Weibull (352.1), log-normal (348.1), and log-logistic (351.1). In the results obtained from the multivariate model were observed three possible risk factors, where the time of treatment to interruption was shorter when dose of BZN ≥6 mg/kg/day (TR 0.55; 95% CI 0.39–0.76) (Fig 1), severe ADRs presented (TR 0.12; 95% CI: 0.07–0.19) (Fig 2) and eosinophilia occurred (TR 0.68; 95% CI: 0.49–0.94) (Fig 3). In addition, a fourth potential risk factor for female patients (TR 0.72; 95% CI: 0.50–1.00) (Fig 4), although this was not strictly statistically significant, a trend was observed that women more often interrupted treatment BZN compared with men (Table 3).

**Table 3:** Risk factors for treatment interruption of benznidazole: Bivariate and multivariate analysis using Generalized Gamma distribution.

	Bivariate analysis				Multivariate analysis			
Covariate	Coefficient (βi)	TR exp(βi)	95% CI	*P*-value	Coefficient (βi)	TR exp(βi)	95% CI	*P*-value
**Severity**								
Mild/Moderate	1.00	1.00			1.00	1.00		
Severe	-2.23	0.11	(0.06–0.18)	**0.000**	-2.10	0.12	(0.07–0.19)	**0.000**
**Doses mg/kg/day**								
< 6	1.00	1.00			1.00	1.00		
≥ 6	-0.68	0.50	(0.27–0.93)	**0.029**	-0.60	0.55	(0.39–0.76)	**0.000**
**Alteration of eosinophil**								
No	1.00	1.00			1.00	1.00		
Yes	-0.67	0.51	(0.27–0.96)	**0.038**	-0.38	0.68	(0.49–0.94)	**0.022**
**Sex**								
Male	1.00	1.00			1.00	1.00		
Female	-0.79	0.45	(0.25–0.80)	**0.007**	-0.33	0.72	(0.50–1.00)	**0.057**
**Age (years)**								
<50	1.00	1.00						
≥50	-0.11	0.90	(0.46–1.77)	0.762				
**ADRs**								
≤ 3	1.00	1.00						
> 3	-0.40	0.67	(0.36–1.25)	0.208				
**History of previous Pathologies**								
No	1.00	1.00						
Yes	-0.12	0.88	(0.44–1.75)	0.734				
**Alteration of leukocytes**								
No	1.00	1.00						
Yes	-0.45	0.96	(0.26–3.38)	0.944				
**Alteration of lymphocyte**								
No	1.00	1.00						
Yes	-0.02	0.97	(0.28–3.43)	0.966				
**Alteration of neutrophils**								
No	1.00	1.00						
Yes	-0.78	0.46	(0.12–1.76)	0.256				

ADRs, adverse drug reactions; Coefficient (βi), β coefficient; TR, time ratio; CI, confidence interval; Bold values, values statistically significant

### Risk factors for severe ADRs

The bivariate analysis showed that female sex (p = 0.013), presence of > 3 ADRs (p = 0.016) and dose (p = 0.001) were statistically significant predictors. Those with a P-value < 0.1 were considered for inclusion in the multivariate model. For the multinomial logistic regression model the best predictors of severe ADRs were: female sex (OR 3.98; 95% CI 1.56–10.16), dose of BZN ≥6 mg/kg/day (OR 1.41; 95% CI 1.17–1.70) and presence of ≥ 3 ADRs (OR 6.47; 95% CI 1.24–34.34) (Table 4).

**Table 4:** Risk factors for severity of adverse drug reactions to benznidazole: Using a multinomial logistic regression model.

		Multivariate analysis			
Severity of ADRs	Covariate	Coefficient (βi)	aOR	95% CI	*P*-value
Mild	Base outcome				
	Female sex	0.60	1.82	(0.96–3.44)	0.065
Moderate	≥ 6mg/kg/day	0.12	1.13	(0.95–1.35)	0.160
	≥ 3 ADRs	1.47	4.35	(1.48–12.78)	0.007
	Female sex	1.38	3.98	(1.56–10.16)	0.004
Severe	≥ 6mg/kg/day	0.34	1.41	(1.17–1.70)	0.000
	≥ 3 ADRs	1.86	6.47	(1.21–34.34)	0.028

ADRs, adverse drug reactions; Coefficient (βi), β coefficient; aOR, adjusted odds ratio; CI, confidence interval.

## Discussion

This study shows that 76.8% of patients treated with Benznidazole completed the full 60-day course of treatment; therefore 23.2% of cases needed definitive treatment interruption. These results contrast with previous studies that have reported a higher percentage of interruption, such as in Brazil, 31% [9,13] and Italy, 32% [11]. Our results also differ with the ones recently published in the largest clinical trial for Chagas disease, where the proportion of permanent treatment discontinuation of BZN was 13.4% in the BZN arm compared with 3.6% in the placebo arm [4]. Other clinical trials also reported lower rates of definitive suspension of BZN. In Brazil, in a clinical trial, unblinded, nonrandomized, the discontinuation rate was 13% [8] and in Argentina in a controlled, multicenter, blinded clinical trial, disruption of BZN was 17.7% due to ADRs [26]. In our study, the average time to interruption was 26.7 days (95% CI: 22.3–34.6 days) and the probability of treatment interruption was highest in the first week, with 23.1% of interruptions. Multivariate analysis shows that treatment interruption was significantly associated with the severity of ADRs, eosinophilia and the dose, followed by female sex, as a fourth possible associated factor, although it was not strictly statistically significant. Meanwhile, female sex, dose were related with severe ADRs.

In the present study, it was evident that severe ADRs predict treatment interruption compared with non-severe ADRs. An association of severity of ADRs with premature interruption had been also previously described for BZN [9]. Of 224 patients who received BZN, 91.5% had at least one ADR. A similar incidence of ADRs, 87.5%, was reported in a previous prospective longitudinal research conducted in Brazil [27]. Others studies had reported lower incidences of 48.7 and 48.9% in Brazil [9,13]. Coinciding with previous findings in adult patients treated with BZN [9,28], this study found that dermatological alterations and gastrointestinal disturbances were predominant.

Out of the identified risk factors, only dose is considered as a potentially modifiable. Indeed, this study shows that a dose of BZN ≥ 6 mg/kg/day is an important predictor of both severe ADRs and treatment interruption. It is also associated with significantly higher incidence of dermatological ADRs, such as rash, itching, urticaria, angioedema, photosensitivity, bullous eruption and skin peeling. Currently there is no published evidence on the consequences of decreasing the dose of BZN on the effectiveness of treatment. Nevertheless, there are a few ongoing studies in this regard [29].

On the other hand, BZN was administered in this study for the recommended duration in ambulatory care (60 days) as most of the treatment regimens in clinical studies and clinical guidelines [10,11,30,31]; therefore, treatment interruption is defined in comparison with that recommendation. However, there are a few studies that have considered a shorter course of treatment [8]. A recent study in chronically *T*. *cruzi* infected patients evaluated a new scheme of BNZ administration with intermittent doses at 5mg/kg/day every 5 days for a total of 60 days (12 days of intermittent treatment). With this new scheme, reported a definite discontinuation in only one patient (1/20). The intermittent treatment of BNZ emerges as a potential solution to improve the safety of administering BNZ [32]. However, it might be of great importance to evaluate their parasitological efficacy compared with the standard duration.

In this study, 9.4% of patients presented eosinophilia during the treatment period without any associated symptoms. Two patients experienced eosinophilia and systemic symptoms—Drug Reaction with Eosinophilia and Systemic Symptoms (DRESS). Previous studies had reported the appearance of this syndrome in 3.7% (3/81) of patients in a cohort treated with Nifurtimox [14]. However, this had not been previously reported for BZN. The presence of eosinophils in the skin is common in dermatological disorders associated with adverse drug reactions and it has been hypothesized that eosinophils might contribute to pathogen defense and regulate inflammatory responses [33,34]. Drugs, either directly or after processing by dendritic cells, activate T cells which can produce cytokines and cytotoxins resulting in tissue damage and an inflammatory response [33,34].

Female sex was found as a predictor of severe ADRs and definitive interruption of BZN (Not statistically significant), a finding that had been previously reported as a strong and independent factor for treatment interruption (OR 2.3; 95% CI: 1.2–4.3) and the most important predictor for overall ADRs (OR 2.9; 95% CI 1.5–5.4) [13]. Recent studies suggest that pharmacokinetic differences between women and men that can impact ADRs development are: lower body weight, lower glomerular filtration rate and higher percentage of body fat [35,36]. Interestingly, for Nifurtimox, no relation with sex has been found in previous studies [37].

The main limitations of this study include a possible information bias and the presence of confounding factors (e.g., other drugs used). Due to its retrospective nature, this study relies on the quality of the original records. This quality can be granted given the standard protocol that was used for treatment indications, lab tests and follow-up over the years. Other measures taken to address the limitations, due to the retrospective nature of the study, were double checking on the review process and a consensus when disagreement. As only five patients (2.2%) were lost to the follow-up, they were not included in the analysis.

Even though the use of BNZ in adults chronically *T*. *cruzi* infected and with established Chagas’ cardiomyopathy showed no significantly reduce cardiac clinical deterioration, the efficacy in adult patients in chronic phase without cardiomyopathy it is still under discussion. One of the most striking problems for assessing its effectiveness and for recommending this drug in clinical practice is the high frequency of ADRs that lead to an important rate of treatment abandonment. In this study, although the frequency of ADRs during administration of BZN was high (91.5%), most of the ADRs were considered as mild and only 23.2% of cases required definitive treatment interruption. As the only modifiable factor related to severe ADRs and treatment interruption was the dose of BZN, a next and needed step is to test the efficacy of BZN at a lower dose. For clinical practice, we recommend the beginning of the clinical controls on the first week of treatment in order to document earlier the number of ADRs before turning into severe and therefore minimize the risk of treatment interruption. Since some of the severe ADRs are of insidious onset, patient awareness could also contribute to avoid or stop some of them.

## Supporting information

S1 File STROBE checklist. (DOCX)

## References

[1] Chagas disease in Latin America: an epidemiological update based on 2010 estimates. Wkly Epidemiol Rec. 2015;90: 33–43. 25671846

[2] Lee BY, Bacon KM, Bottazzi ME, Hotez PJ. Global economic burden of Chagas disease: a computational simulation model. Lancet Infect Dis. 2013;13: 342–8. doi: 10.1016/S1473-3099(13)70002-1 23395248 10.1016/S1473-3099(13)70002-1 PMC3763184

[3] Croft SL, Barrett MP, Urbina JA. Chemotherapy of trypanosomiases and leishmaniasis. Trends Parasitol. 2005;21: 508–512. doi: 10.1016/j.pt.2005.08.026 16150644 10.1016/j.pt.2005.08.026

[4] Morillo CA, Marin-Neto JA, Avezum A, Sosa-Estani S, Rassi A, Rosas F, et al Randomized Trial of Benznidazole for Chronic Chagas’ Cardiomyopathy. N Engl J Med. 2015;373: 1295–306. doi: 10.1056/NEJMoa1507574 26323937 10.1056/NEJMoa1507574

[5] Villar JC, Perez JG, Cortes OL, Riarte A, Pepper M, Marin-Neto JA, et al Trypanocidal drugs for chronic asymptomatic Trypanosoma cruzi infection. Cochrane database Syst Rev. 2014;5: CD003463 doi: 10.1002/14651858.CD003463.pub2 24867876 10.1002/14651858.CD003463.pub2 PMC7154579

[6] Fabbro De Suasnábar D, Arias E, Streiger M, Piacenza M, Ingaramo M, Del Barco M, et al Evolutive behavior towards cardiomyopathy of treated (nifurtimox or benznidazole) and untreated chronic chagasic patients. Rev Inst Med Trop Sao Paulo. 2000;42: 99–109. 10810325 10.1590/s0036-46652000000200007

[7] Cancado JR. Long term evaluation of etiological treatment of chagas disease with benznidazole. Rev Inst Med Trop Sao Paulo. 2002;44: 29–37. 11896410

[8] Viotti R, Vigliano C, Lococo B, Bertocchi G, Petti M, Alvarez MG, et al Long-term cardiac outcomes of treating chronic Chagas disease with benznidazole versus no treatment: a nonrandomized trial. Ann Intern Med. 2006;144: 724–34. 144/10/724 [pii] 16702588 10.7326/0003-4819-144-10-200605160-00006

[9] Hasslocher-Moreno AM, do Brasil PEAA, de Sousa AS, Xavier SS, Chambela MC, Sperandio da Silva GM. Safety of benznidazole use in the treatment of chronic Chagas’ disease. J Antimicrob Chemother. 2012;67: 1261–1266. doi: 10.1093/jac/dks027 22331592 10.1093/jac/dks027

[10] Miller DA, Hernandez S, Rodriguez De Armas L, Eells SJ, Traina MM, Miller LG, et al Tolerance of benznidazole in a United States Chagas Disease clinic. Clin Infect Dis An Off Publ Infect Dis Soc Am. 2015;60: 1237–1240. doi: 10.1093/cid/civ005 25601454 10.1093/cid/civ005

[11] Antinori S, Grande R, Bianco R, Traversi L, Cogliati C, Torzillo D, et al High frequency of adverse reactions and discontinuation with benznidazole treatment for chronic Chagas disease in Milan, Italy. Clin Infect Dis An Off Publ Infect Dis Soc Am. 2015;60: 1873–1875. doi: 10.1093/cid/civ230 25805302 10.1093/cid/civ230

[12] Altcheh J, Moscatelli G, Moroni S, Garcia-Bournissen F, Freilij H. Adverse events after the use of benznidazole in infants and children with Chagas disease. Pediatrics. 2011;127: e212–218. doi: 10.1542/peds.2010-1172 21173000 10.1542/peds.2010-1172

[13] Sperandio da Silva GM, Mediano MFF, Alvarenga Americano do Brasil PE, da Costa Chambela M, da Silva JA, de Sousa AS, et al A clinical adverse drug reaction prediction model for patients with chagas disease treated with benznidazole. Antimicrob Agents Chemother. 2014;58: 6371–6377. doi: 10.1128/AAC.02842-14 25114135 10.1128/AAC.02842-14 PMC4249401

[14] Jackson Y, Alirol E, Getaz L, Wolff H, Combescure C, Chappuis F. Tolerance and safety of nifurtimox in patients with chronic chagas disease. Clin Infect Dis an Off Publ Infect Dis Soc Am. 2010;51: e69–75. doi: 10.1086/656917 20932171 10.1086/656917

[15] Rassi A, Rassi A, Marin-Neto JA. Chagas disease. Lancet. 2010;375: 1388–1402. doi: 10.1016/S0140-6736(10)60061-X 20399979 10.1016/S0140-6736(10)60061-X

[16] Ministerio de la Protección Social. Guía para la atención clínica integral del paciente con enfermedad de Chagas [Internet]. Bogotá, República de Colombia; 2010 Available: https://www.minsalud.gov.co/Documentos%20y%20Publicaciones/Guia%20de%20atencion%20clinica%20de%20chagas%202010.pdf

[17] Naranjo CA, Busto U, Sellers EM, Sandor P, Ruiz I, Roberts EA, et al A method for estimating the probability of adverse drug reactions. Clin Pharmacol Ther. 1981;30: 239–245. 7249508 10.1038/clpt.1981.154

[18] Coelho HL, Arrais PS, Gomes AP. Ceara State Pharmacovigilance System: a year of experience.Cad Saude Publica. 1999;15: 631–640. 10502160 10.1590/s0102-311x1999000300021

[19] Landis JR, Koch GG. The measurement of observer agreement for categorical data. Biometrics. 1977;33: 159–174. 843571

[20] Ng ES-W, Klungel OH, Groenwold RHH, van Staa T-P. Risk patterns in drug safety study using relative times by accelerated failure time models when proportional hazards assumption is questionable: an illustrative case study of cancer risk of patients on glucose-lowering therapies. Pharm Stat. 2015; doi: 10.1002/pst.1697 26123413 10.1002/pst.1697

[21] Cox C, Chu H, Schneider MF, Muñoz A. Parametric survival analysis and taxonomy of hazard functions for the generalized gamma distribution. Stat Med. 2007;26: 4352–4374. doi: 10.1002/sim.2836 17342754 10.1002/sim.2836

[22] Lee ET, Wang JW. Statistical Methods for Survival Data Analysis. New Jersey: John Wiley & Sons; 2003.

[23] Kleinbaum DG, Klein M. Survival Analysis–A Self-Learning Text. New York: Springer; 2005.

[24] Hosmer DW, Lemeshow S. Applied logistic regression, 2nd ed. New York: Wiley & Sons; 2000.

[25] R Development Core Team. A language and environment for statistical computing Vienna, Austria: R Foundation for Statistical Computing; 2013.

[26] Sosa-Estani S, Armenti A, Araujo G, Viotti R, Lococo B, Ruiz Vera B, et al [Treatment of Chagas disease with benznidazole and thioctic acid]. Medicina (B Aires). Argentina; 2004;64: 1–6. 15034949

[27] Pontes VMO De, Souza Júnior AS De, Cruz FMT Da, Coelho HLL, Dias ATN, Coêlho ICB, et al Reações adversas em pacientes com doença de Chagas tratados com benzonidazol, no Estado do Ceará. Rev Soc Bras Med Trop. 2010;43: 182–187. doi: 10.1590/S0037-86822010000200015 20464150 10.1590/s0037-86822010000200015

[28] Carrilero B, Murcia L, Martínez-Lage L, Segovia M. Side effects of benznidazole treatment in a cohort of patients with Chagas disease in non-endemic country. Rev Española Quimioter Publicación Of La Soc Española Quimioter. 2011;24: 123–126. 21947093

[29] DNDi. New Benznidazole Regimens / Combos—DNDi [Internet]. 2015. Available: https://www.dndi.org/diseases-projects/portfolio/new-benz-regimens/

[30] Pinazo M-J, Muñoz J, Posada E, López-Chejade P, Gállego M, Ayala E, et al Tolerance of benznidazole in treatment of Chagas’ disease in adults. Antimicrob Agents Chemother. 2010;54: 4896–4899. doi: 10.1128/AAC.00537-10 20823286 10.1128/AAC.00537-10 PMC2976114

[31] Olivera MJ, Fory JA, Olivera AJ. Quality assessment of clinical practice guidelinesfor Chagas disease. Rev Soc Bras Med Trop. 2015;48: 343–346. doi: 10.1590/0037-8682-0251-2014 26108016 10.1590/0037-8682-0251-2014

[32] Alvarez MG, Hernandez Y, Bertocchi G, Fernandez M, Lococo B, Ramirez JC, et al New Scheme of Intermittent Benznidazole Administration in Patients Chronically Infected with Trypanosoma cruzi: a Pilot Short-Term Follow-Up Study with Adult Patients. Antimicrob Agents Chemother. 2015;60: 833–837. doi: 10.1128/AAC.00745-15 26596935 10.1128/AAC.00745-15 PMC4750658

[33] de Graauw E, Beltraminelli H, Simon H-U, Simon D. Eosinophilia in Dermatologic Disorders. Immunol Allergy Clin North Am. 2015;35: 545–560. doi: 10.1016/j.iac.2015.05.005 26209899 10.1016/j.iac.2015.05.005

[34] White KD, Chung W-H, Hung S-I, Mallal S, Phillips EJ. Evolving models of the immunopathogenesis of T cell–mediated drug allergy: The role of host, pathogens, and drug response. J Allergy Clin Immunol. 2015;136: 219–234. doi: 10.1016/j.jaci.2015.05.050 26254049 10.1016/j.jaci.2015.05.050 PMC4577472

[35] Meibohm B, Beierle I, Derendorf H. How important are gender differences in pharmacokinetics? Clin Pharmacokinet. 2002;41: 329–342. doi: 10.2165/00003088-200241050-00002 12036391 10.2165/00003088-200241050-00002

[36] Anderson GD. Chapter 1 Gender Differences in Pharmacological Response. International review of neurobiology. 2008 pp. 1–10. doi: 10.1016/S0074-7742(08)00001-9 10.1016/S0074-7742(08)00001-9 18929073

[37] Olivera MJ, Cucunuba ZM, Alvarez CA, Nicholls RS. Safety Profile of Nifurtimox and Treatment Interruption for Chronic Chagas Disease in Colombian Adults. Am J Trop Med Hyg. 2015;93: 1224–1230. doi: 10.4269/ajtmh.15-0256 26392162 10.4269/ajtmh.15-0256 PMC4674239

